# Recent Advances in Electrochemical Biosensors for Food Control

**DOI:** 10.3390/mi14071412

**Published:** 2023-07-13

**Authors:** Francesco Rizzotto, Majd Khalife, Yanxia Hou, Carole Chaix, Florence Lagarde, Natale Scaramozzino, Jasmina Vidic

**Affiliations:** 1 Université Paris-Saclay, INRAE, AgroParisTech, Micalis Institute, 78350 Jouy en Josas, France; 2 University Grenoble Alpes, CEA, CNRS, IRIG-SYMMES, 38000 Grenoble, France; 3 University Lyon, CNRS, University Claude Bernard Lyon 1, Institute of Analytical Sciences, 5 Rue de la Doua, 69100 Villeurbanne, France; 4 University Grenoble Alpes, CNRS, LIPhy, 38000 Grenoble, France

**Keywords:** electrochemical sensor, food safety, detection, genosensor, aptasensor, immunosensor

## Abstract

The rapid and sensitive detection of food contaminants is becoming increasingly important for timely prevention and treatment of foodborne disease. In this review, we discuss recent developments of electrochemical biosensors as facile, rapid, sensitive, and user-friendly analytical devices and their applications in food safety analysis, owing to the analytical characteristics of electrochemical detection and to advances in the design and production of bioreceptors (antibodies, DNA, aptamers, peptides, molecular imprinted polymers, enzymes, bacteriophages, etc.). They can offer a low limit of detection required for food contaminants such as allergens, pesticides, antibiotic traces, toxins, bacteria, etc. We provide an overview of a broad range of electrochemical biosensing designs and consider future opportunities for this technology in food control.

## 1. Introduction

Food safety is a major public health concern, and its control represents a priority for the food supply chain. The World Health Organization (WHO) indicated around 200 different types of disease caused by eating contaminated food [1]. In some cases, foodborne and waterborne diseases cause long-term health problems and even death, especially in vulnerable populations such as pregnant women, newborns, and elderly people. Moreover, approximately USD 110 billion is lost every year in productivity and medical expenses caused by food intoxications, making food safety an important economic issue, too [2]. In particular, the low-income countries face serious problems of food safety due to the food storage and distribution at environmental temperature, without strict hygienic control, together with inadequate surveillance systems and the lack of infrastructure for food analysis [2,3,4]. In developed countries, the increased use of ready-to-eat (RTE) foods and beverages that are consumed without any further processing significantly increases the risk of food intoxication, which underlines the need of strict food control measures [5].

**Figure 1:**
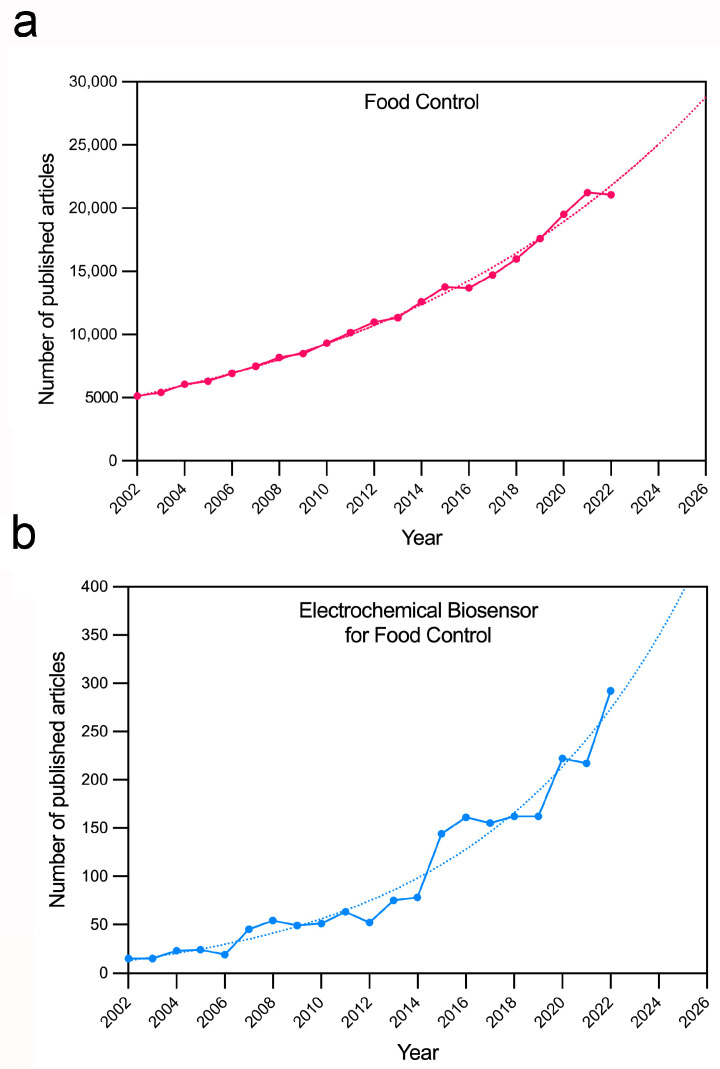
Food control and electrochemical biosensors in the literature in the period of 2002–2022. (**a**) Values were obtained by searching “food control” and (**b**) “electrochemical biosensors for food control” in Scopus (solid lines). Trends obtained by fitting a tendency curve and projecting it for the next 4 years (dotted lines).

The global incidence of foodborne disease is difficult to estimate, but the European Food Safety Authority and the European Centre for Disease Prevention and Control have reported about 205,202 hospitalizations due to confirmed zoonoses with about 350 fatal cases in 2021. The WHO has estimated 600 million annual deaths worldwide due to unsafe food [6]. The real incidence of foodborne illnesses and outbreaks is underestimated because of misdiagnosis, under-reporting, and improper examination. Figure 1a shows evolution of the number of articles published for “food control” in the last twenty years and the future trend. Food contaminants can be classified as biological, physical, or chemical. All three categories are connected since a single food hazard frequently introduces other types of contamination. In the food risk assessment, a biological hazard is a biological agent, as are bacteria and toxins, with the potential to cause an adverse human health effect when present into an edible product. Among foodborne pathogens, *Listeria*, *Salmonella*, *Campylobacter* spp., Norovirus, and Shiga toxin-producing *Escherichia coli* are responsible for the vast majority of illnesses, hospitalizations, and deaths. Chemical hazard occurs when chemicals are present in food at a harmful level for humans. Within the broad family of chemical food contaminants, disinfectants, endocrine hormones disrupting chemicals, fragrances, pesticides, fluorinated substances, and pharmaceuticals, such as antibiotics, can be listed as synthetic chemicals that may cause ecological or human health risks. The most common physical contaminants are micro- and nanoplastics along with different nanoparticles from packaging or wraps, broken glasses, and dirt from unwashed fruits and vegetables.

Conventional analytical techniques for chemical and physical contaminants are mostly based on chromatographic techniques and mass spectrometry. These technologies are accurate but can only be carried out by specialized labs equipped with state-of-the-art instrumentation, which impairs the capacity of continuous control. Moreover, these methods suffer from drawbacks such as matrix interferences and high costs; they are based on time- and resource-intensive analysis and require highly trained personnel. Likewise, biological food safety analysis necessarily passes through centralized laboratory facilities, entailing high costs, highly experienced experts and an impracticability of an on-site monitoring. Both currently used traditional and molecular methods for foodborne pathogen detection suffer from some limitations. Traditional methods are culture-based and involve the growth of bacteria on specific media for enrichment, isolation, and identification [7,8]. Although these methods are robust, they take a long time to provide results (up to one week), have a high risk of contamination and confirmation tests are usually required. Moreover, some pathogens, such as *Listeria*, *Salmonella*, and *Campylobacter*, can remain in viable but nonculturable (VBNC) form and cannot be detected by culture-based methods. Molecular methods, based on the use of polymerase chain reaction (PCR) detect DNA/RNA of targeted pathogens [9,10,11]. PCR-based methods are effective but laborious and may give false negative results because of the sensitivity of the polymerase enzyme to food compounds, which limits their efficiency [11]. Furthermore, molecular methods may give false positive results when DNA of dead microorganisms is evidenced in food, which does not entail any risk for consumers [10].

The WHO defined the criteria that should be satisfied in the development of new analytical tools and summarized them in the acronym REASSURED (Real-time connectivity, Ease of specimen collection, Affordable, Sensitive, Specific, User-friendly, Rapid and robust, Equipment-free, and Deliverable to end-users) [12]. In such a context, a growing interest in biosensors has been observed in recent years, since they offer low-cost analysis, high specificity and sensitivity, easy implementation and miniaturization, thus showing high potential for meeting the REASSURED criteria. Biosensors are analytical devices that associate a bioreceptor, like nucleic acid, aptamer, or antibody, to an active transducer that transforms the recognition event into a measurable signal for the detection of chemical or biological analytes [13]. Among them, electrochemical biosensors may exhibit highly versatile detection schemes, real-time quantification, and label-free and multiplex detection, providing a promising tool for food safety control [14]. Figure 1b shows the evolution of the number of articles published for “electrochemical biosensors for food safety” in the last twenty years and its future trend. The development of electrochemical biosensors is particularly favored due to the availability of low-cost and small-size disposable electrodes compatible with point-of-need and on-site analytical monitoring [9,15,16].

In this review, the most recent electrochemical biosensors developed for the detection of pathogens and contaminants in food are described. We present an overview of the current approaches with interest for food safety applications. It is noteworthy that application of nanomaterials in electrochemical biosensors, electrode modifications, and device integration have been comprehensively described in some recent reviews [17,18,19,20]. Here, we focus on progress in different strategies applied in electrochemical detection, such as biomolecule interaction, enzymatic and nanoparticle signal amplification, multiplexing, and continuous monitoring. We also cover future perspectives and challenges.

## 2. Electrochemical Biosensors

**Figure 2:**
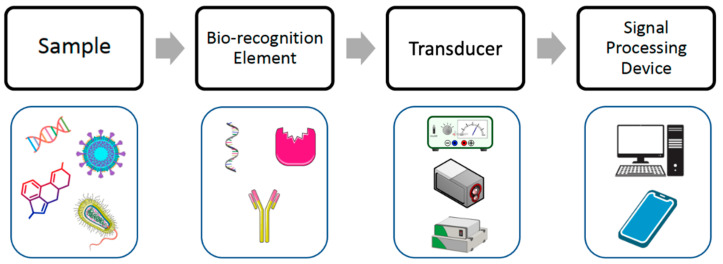
Different elements and steps in electrochemical detection of foodborne contaminants. Food sample containing biological or chemical hazardous elements in contact with the biorecognition element immobilized on the electrode generates an electrical signal that can be processed by a computer or a smartphone.

**Figure 3:**
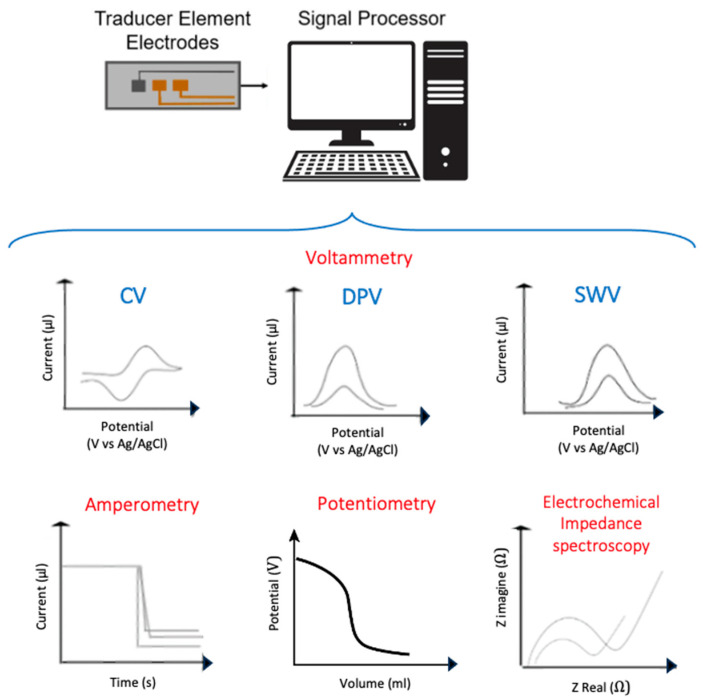
Different electrochemical methods that enable to evidence and quantify foodborne contaminants using electrochemical biosensors.

Electrochemistry studies the electrical parameters related to chemical reactions, analyzing the electricity as an outcome of a chemical process. An electrochemical biosensor is based on an electrochemical transducer, capable of providing selective and quantitative analytical information on the electron transfer variations due to interaction between the analyte and the bioreceptor [21,22], as illustrated in Figure 2. Bioreceptors serve as a recognition element that binds or transforms the analyte, while the electrochemical transducer converts the event of bioreceptor–analyte interaction into an electrical signal. Different electrochemical techniques such as potentiometry, voltammetry, impedance, and amperometry are employed in biosensors (Figure 3). In potentiometry, the potential of an electrochemical cell under static conditions is measured, holding the current constant. In amperometry, the potential of the electrode is held constant. In impedance techniques, such as the widely used electrochemical impedance spectroscopy (EIS), the relationship between the alternative current and the applied sinusoidal potential in a frequency domain is measured [23]. In voltammetry, such as differential potential voltammetry (DPV), square wave voltammetry (SWV), cyclic voltammetry (CV), or linear sweep voltammetry (LSV), the current change is analyzed under controlled but not constant potential [17,24].

Methods for immobilizing bioreceptors to electrode surfaces have been extensively studied aiming at both providing an intimate contact between the recognition entities and the surface of the electrode and keeping the activity of bioreceptor. Gold and carbon-based materials (i.e., single-wall, multiwall carbon nanotubes, and graphene) are mostly used electrode materials in biosensors because they can be functionalized with biological molecules highly efficiently in a simple way. In addition, they are compatible with chemically and biologically active molecules and do not interfere with the recognition event. Moreover, these surfaces can be easily modified with other materials, such as nanoparticles, giving upgraded transducers with improved sensitivity and increased loading capacities [17]. Other frequently used materials for electrode fabrication are metals (platinum, silver), ceramic (indium tin oxide, titanium oxide, polysilicon) and polymers (poly(acetylene), poly(pyrrole), poly(aniline), chitosan). The silica nanoparticles showing a great biocompatibility, tunable pore structure, and excellent uniformity also provide benefits to electrochemical sensing platforms [25].

The immobilization of the bioreceptor is a key step in electrochemical biosensor development because its performances, such as sensitivity, robustness, and stability, directly depends on the orientation and conformation of the recognition element and surface density. The immobilization of bioreceptors is usually achieved by covalent interactions (such as gold–thiol interaction or amino link), affinity binding (such as biotin–streptavidin binding), adsorption, or entrapment. Adsorption is the simplest method, requiring no specific reagents or bioreceptor modifications but is the least stable and cannot provide uniform orientation of molecules on the electrode surface. Blocking step is usually performed before detection to prevent nonspecific absorption of the analyte or matrix component on the electrode. For instance, 6-mercapto-1-hexanol as a blocking agent was shown to control surface properties of immobilized thiolate–DNA probe on gold surface, serving as a spacer between attached probes, and preventing nonspecific DNA–gold surface interaction [26]. Albumin is a blocking agent frequently used in immunosensors.

Amplification of the electrochemical signal can be achieved by using highly conductive nanomaterials conjugated with well-oriented bioreceptors or combined with specific labeling. For instance, various electroactive species such as metallic and semiconductor nanoparticles loaded onto the electrode surface significantly amplify the electrical signal. Enzymes associated with functionalized electrodes sensitively boost redox cycling and provide signal enhancement [27].

Bioreceptors used in electrochemical sensors can be natural, bioinspired, and biomimetic. Natural bioreceptors can be found in living organisms, and the most commonly used are antibodies, enzymes, nucleic acids (DNA and RNA), bacteriophages, and whole cells, membranes, and organelles. Natural bioreceptors have inherent high specificity. However, they can be unstable under nonphysiological conditions, and their purity/activity may vary from batch to batch. Bioinspired molecules (e.g., aptamers, nanobodies, peptides) are synthetically derivatives from natural molecules obtained through rational engineering. Molecularly imprinted polymers (MIPs) are examples of biomimetic receptors. Bioreceptors of the two latter categories offer enhanced stability compared to natural receptors. Due to their enhanced affinity toward the target of the analysis (similar to antibodies), they provide the biosensor with the selectivity required for highly sensitive and specific detection [28].

Table 1 summarizes some recent electrochemical biosensors using various recognition elements and transduction systems together with their performances. Clearly, the analytical performances of the electrochemical biosensors are remarkable compared to classical analytical methods. The complexity of the food matrix still represents a challenge for the food control diagnostics. Different foods are highly divers in pH, density, and composition (X1). While tap water does not present particular complications except for the presence of some ions, other liquid matrices such as juices may affect the analysis, mainly due to their pH. Milk, is characterized as having a high amount of proteins and fats, whereas fruit and vegetables can be difficult to analyze due to the presence of organic acids and antimicrobial compounds. Meat is a protein-rich food source with low carbohydrate content, abundant in oligo-element like iron, selenium, vitamins, and folic acid; all may be electroactive species. Meat is also characterized by having a complex structure consisting of a myofibrillar protein system. Considering such matrix complexity in food samples, an electrochemical biosensor needs to have extremely high affinity and selectivity to detect a contaminant with low abundance.

### 2.1. Electrochemical Genosensors

Electrochemical genosensors, also called nucleic-acid-based biosensors, enable rapid and accurate food quality control diagnostics through detection of specific DNA or RNA sequences. Typically, they detect DNA hybridization when a target DNA is recognized by the immobilized single-stranded DNA (ssDNA) probe, which results in the formation of a double-stranded hybrid DNA (dsDNA). Taking into account that DNA is more resistant to food processing procedures than proteins, targeting specific DNA is a promising tool for adulteration analysis, detection of genetically modified organisms (GMOs), or allergenic proteins and peptides [150,151,152]. The production of DNA probes that ensure recognition is faster and more accessible than the production of other bioreceptors, which makes the construction of the biosensor relatively simple and affordable [22]. Unlabeled DNA probes can be efficiently cross-linked with paper-based electrodes when exposed to the UV light [153]. Alternatively, DNA probes can be produced with chemical modification at their 3′- or 5′- ends for their efficient grafting onto the electrode. For instance, DNA probes carrying thiol, biotin or amino groups are easily attached to the electrode surface carrying gold, streptavidin, or a carboxyl group, respectively [16].

The conversion of the hybridization between the target nucleic acid sequences and the immobilized DNA probe into an electrical signal usually requires the use of DNA intercalants or other redox active molecules. The most used DNA intercalants are methylene blue, a two-electron redox molecule that covalently binds guanine bases in ssDNA, [Os(bpy)_2_dppz]^2+^ (dppz = dipyrido[3,2-a:2′,3′-c]phenazine), a reversible one-electron oxidizing metal complex that strongly binds to dsDNA, and Ru(NH_3_)_6_^3+^, a reversible one-electron metal complex that electrostatically interacts with the anionic DNA backbone [154,155]. The most used nonintercalant redox indicators are potassium ferro/ferricyanide, ferrocene, Nile blue, Toluidine blue, ethidium bromide, and tripropylamine [24,156]. Razmi et al. [32] developed a highly sensitive electrochemical genosensor, for the detection of *E. coli* O157:H7 in environmental water. To increase the electrode conductivity and the surface area available for the immobilization of the probe, gold nanostars were deposited onto a gold electrode. A thiolate ssDNA probe, designed to specifically target *E. coli* O157:H7, was then immobilized onto the electrode surface and labeled with the electroactive toluidine blue by drop-casting method. The authors reported a linear concentration range of 7.3 to 1 × 10^−17^ µM in environmental water samples, with a limit of detection (LOD) as low as 0.01 zM. Although extremely sensitive, this label-based genosensor showed limited selectivity and variability at lower bacterial concentrations or under pH variation.

**Figure 4:**
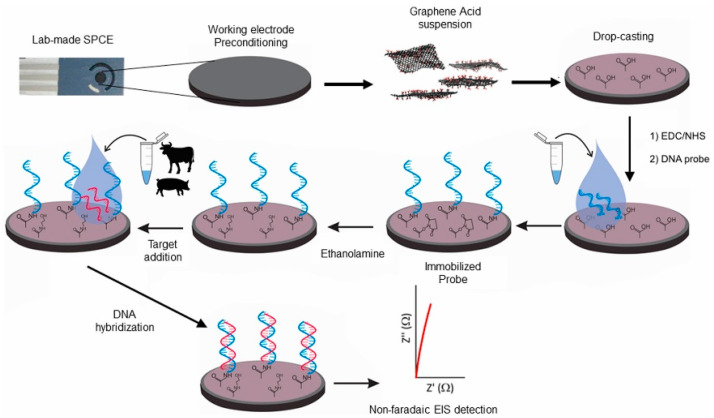
Functionalization of screen-printed carbon electrode with a graphene acid suspension to enable a specific ssDNA grafting for sensing of pork DNA using the nonfaradaic EIS. Adapted with permission from [150].

Nonintercalant redox molecules are not considered as a labeling, but their utilization in genosensors increases the price and complexity of the analysis. Electrochemical enzyme-linked detection of DNA hybridization employs enzymes, such as alkaline phosphatase, peroxidase, or glucose oxidase in combination with their appropriate substrates. In these sensors, electrochemically active indicator is produced by enzymatic conversion of the substrate [157]. This strategy results in signal amplification due to the ability of the enzyme to convert many molecules of the substrate. Finally, some label-free electrochemical genosensors that do not require the introduction of redox active molecule in the food matrix were also reported. For instance, DNA probe was immobilized on a screen-printed carbon electrode modified with graphene acid to improve the charge transport properties of the electrode [150]. The presence of pork mitochondrial DNA was detected in beef samples using nonfaradaic EIS without the need of redox indicators (Figure 4).

Recently, Somayeh et al. [47] proposed an alternative genosensing approach for the detection of *Staphylococcus aureus* in milk. In their assay, a specific extracellular endoexonuclease of *S. aureus*, micrococcal nuclease, was detected using a DNA probe unrelated to the bacterium. To do this, they created a U-shaped DNA structure on the gold electrode surface by immobilizing two oligonucleotide sequences, and subsequently drop-casted complementary sequences. The formation of the dsDNA prevented the redox indicators to access the gold surface, thereby providing a low baseline electrochemical signal. In the presence of micrococcal nuclease, the hydrolyzation of the oligonucleotides facilitated the redox indicator to reach the surface, dramatically increasing the signal. This electrochemical biosensor had a dynamic range from 0.0002 U/µL to 0.0033 U/µL and a LOD of 2.15 × 10^−5^ U/µL in spiked milk samples. However, the test showed limits for a rapid analysis when samples contaminated with *S. aureus* were cocontaminated with other bacteria, such as *E. coli*, that also produced the micrococcal nuclease enzyme.

### 2.2. Electrochemical Immunosensors

Antibodies are molecules naturally present in the serum of vertebrate organisms and play a fundamental role in the defense mechanism that constitutes the acquired immune system. They have a direct recognition ability characterized by the excellent specificity for some antigens that can be present in food such as proteins, peptides, nucleic acids, nanoplastics and toxins. This is why a number of electrochemical immunosensors have been proposed (Table 1). However, the analytical properties of immunosensors are limited by the stability of antibodies under the storage and working conditions employed. When applied to bacterial detection, antibodies cannot distinguish between live and dead cells [158].

To obtain well-ordered layers of antibodies on the electrode surface, usually a self-assembled monolayers (SAM) immobilization method is used [16,159,160]. Thiol SAMs-modified gold electrodes are the most reported substrates in electrochemical immunosensors. Some nanomaterials, such as carboxyl graphene, which exposes the carboxylic acid and phenolic hydroxyl groups when dispersed in water, gave a possibility to covalently graft antibodies via amide or ester linkages [161]. Most impedimetric immunosensors are in label-free format, while other electrochemical methods require redox indicators and/or amplification of the antibody-antigen interaction signal. Redox indicator may be associated with the electrode material to simplify the detection procedure, as it was reported for influenza virus detection using ferrocene-bearing polypyrrole electrode [162,163].

Other attractive materials for electrode functionalization are metal-organic frameworks (MOF) with large surface area, high conductivity, and good stability. A label-free electrochemical immunosensor based on (MOF)-derived carbon material, gold nanoparticles (AuNPs)/Zn/Ni-ZIF-8-800@Graphene, was developed for the detection of aflatoxin B1 in peanut oil [72]. Carcinogenic Aflatoxin B1, produced by the molds *Aspergillus flavus* and *A. parasitica*, is the strongest mycotoxin among different types of aflatoxins that stay stable even at the temperature above 100 °C. To achieve its sensitive detection, the authors modified the glassy carbon electrode with bimetallic organic framework material (Zn/Ni-ZIF-8-800), chitosan, and gold nanoparticles. Chitosan was used as an efficient dispersant of graphene, since it has excellent film-forming ability and good adhesion while AuNPs were added to increase electrode conductivity, biocompatibility, and for direct immobilization of the monoclonal antiaflatoxin antibody. The obtained electrochemical sensor showed low experimental cost, high selectivity, and long-term stability. Under the optimal conditions, its linear range was between 0.18–100 ng/mL of aflatoxin B1, and the detection limit was 0.18 ng/mL.

**Figure 5:**
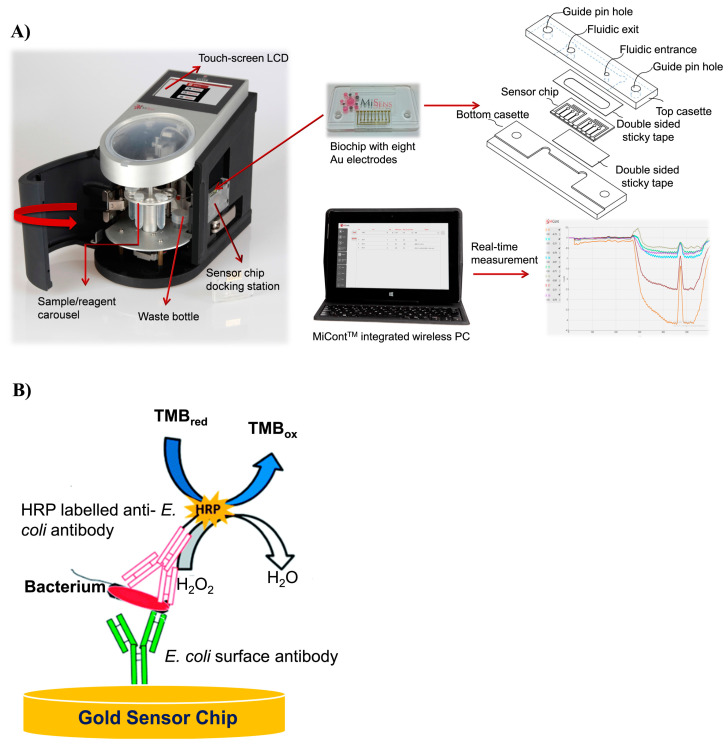
(**A**) Illustration of biochip integrating the microfluidics and electronic connection for biosensor. (**B**) *E. coli* detection strategy, adapted with permission from [164].

An electrochemical immunosensor can be integrated into a microfluidic system for a real-time electrochemical measurement. For this, a design of the biochip is highly important because the fabrication of the electrode array integrating the microfluidics and electronic connections is usually expensive, time-consuming, and labor-intensive. Altintas et al. proposed such a fully automated microfluidic-based electrochemical biosensor for *E. coli* detection [164]. A specific anti-*E. coli* antibody was immobilized using the SAM method on the electrode to capture bacterial cells from the sample. Captured cells were electrochemically detected after the addition of a horseradish peroxidase-labeled anti-*E. coli* antibody and its substrate 3,3′,5,5′-tetramethylbenzidine (TMB)/H_2_O_2_ (Figure 5). The cost of the system can be significantly reduced through the electrode surface multiple regeneration using an agent that breaks antibody–antigen interaction.

### 2.3. Electrochemical Sensors Based on Artificial Bioreceptors

Aptamers, peptides, and molecular imprinted polymers are attractive bioreceptors for pathogen detection because they target the entire microorganism, which enormously simplifies the detection protocols [24,28,165,166]. Sample preparation is the most time-consuming and expensive step in most protocols for foodborne pathogen detection [9]. In addition, artificial bioreceptors may target a variety of other food contaminates such as toxins, allergens, or micro-nano-plastics.

Aptamers are single-stranded oligonucleotides (RNA or DNA), able to recognize and bind a large variety of targets including toxins, metals, drugs, and pathogens. They are selected by Systematic Evolution of Ligands by Exponential Enrichment (SELEX) for their morphological affinity for specific targets, similarly to those of antibodies. The development of electrochemical biosensors implemented with aptamer technology has seen an important growth in recent years, due to their promising advantages, compared to antibodies, such as high tolerance and chemical stability, low-cost production, and easy chemical modifications [167]. Yuan et al. [115] developed an electrochemical aptasensor for the simultaneous detection of Pb^2+^ and Cd^2+^ ions in fruits and vegetables. For this, DNA sequences, complementary to the aptamers specific for Pb^2+^ and Cd^2+^ ions, were immobilized on an electrode surface. When aptamers, previously labelled with methylene blue and ferrocene as redox probes, interacted with immobilized complementary probes, dsDNA were formed onto electrode surface. Upon drop-casting of orange or lettuce samples containing Pb^2+^ and Cd^2+^ ions, aptamers dissociated from the complementary sequences; therefore, the redox probes were removed from the electrode surface, provoking a reduction of the signal. The sensor allowed a detection with a linear range from 0.1 to 1000 nmol/mL and a LOD of 89.31 pmol/L and 16.44 pmol/L for Cd^2+^ and Pb^2+^ ions, respectively.

Svigelj et al. [101] reported an electrochemical aptasensor for gluten screening in gluten-free beer and soy sauce. Gluten is a two-component molecule composed of gliadin and glutenin proteins. Thiolate aptamers, selected for their affinity to gliadin, were immobilized on AuNPs deposited on a screen-printed carbon electrode. The gliadin binding to the aptamer was directly detected in a label-free impedance assay with a LOD of 0.05 mg/L, corresponding to 0.1 mg/L of gluten. This sensor was successfully applied for the analysis of real food containing gluten in its hydrolyzed form.

Click chemistry reaction as an immobilization or sensing strategy in electrochemical aptasensors is also highly attractive. One of the most used common click reactions is the Cu^+^-catalyzed alkyne-azide cycloaddition (CuAAC). Wei et al. [168] applied dual signal amplification of Cu_3_(PO_4_)_2_-mediated click chemistry and DNAzymes to quantify *Salmonella typhimurium* with high accuracy. Bacterial cells were captured in a sandwich between aptamer-modified magnetic beads and Concavalin A-Cu_3_(PO_4_)_2_ hybrid nanoflowers. Selected aptamers recognized specific epitopes on the *S. typhimurium* surface, while Concavalin from the hybrid nanoflowers recognized O-antigen present on the bacterial surface. After bacterial capturing, Cu_3_(PO_4_)_2_ from the hybrid nanoflowers was dissolved with EDTA to release Cu^2+^, which was further reduced to Cu^+^ that triggered a CuAAC reaction on the electrode surface. The electrical signal was amplified by DNAzymes immobilized onto the gold electrode. A detection limit of 10 CFU/mL was obtained using the assay.

**Figure 6:**
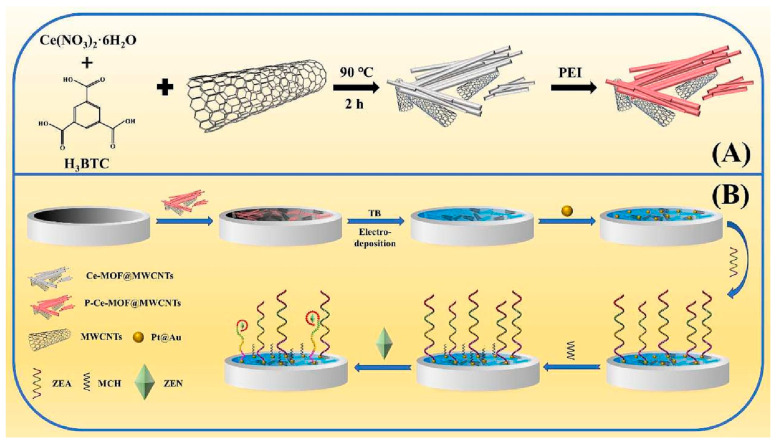
Illustration of synthesis of P-Ce-MOF@MWCNTs (**A**) and stepwise construction of the electrochemical aptasensor for trace detection of zearalenone (**B**). Adapted with permission from [96].

Very recently, Lai et al. [96] presented a signal-off electrochemical aptasensor for the detection of zearalenone mycotoxin. The sensor was constructed using a functionalized nanocomposite of Ce-based metal-organic framework and multiwalled carbon nanotubes to obtain large surface area and high electrochemical activity (Figure 6). To enhance the signal response, the redox probe toluidine blue was bound to aptamers, while Platinum@Au nanoparticles were used to immobilize specific aptamers. When zearalenone was present in the sample, aptamers dissociated from the electrode surface to bind to it, leading to a decrease in the electrochemical signal generated by toluidine blue. The aptasensor exhibited a linear range of 5.0 × 10^−5^ to 50.0 ng/mL, with a LOD of 1.0 × 10^−5^ ng/mL in buffer. The developed aptasensor was successfully applied to detect zearalenone in semen coccis powder.

Although aptamers present high potential as recognition elements in electrochemical biosensors, they can be degraded by nucleases present in the food matrix, compromising the biosensor stability. Moreover, food matrix may modify their 3D structure, which can drastically decrease their recognition efficiency when applied in-field. Finally, aptamers may nonspecifically bind different molecules in complex samples, increasing the challenges for an accurate detection and quantification of the target. In some cases, these drawbacks can be eliminated by the sample preparation prior to analysis, such as EDTA treatment, or targets magnetic preconcentration and washing [167,169,170].

**Figure 7:**
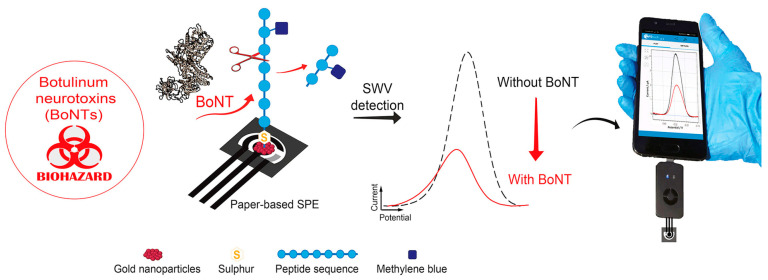
Botulinum neurotoxins detection using the peptide-based electrochemical biosensor. Thiolate peptide carrying methylene blue was grafted on AuNPs deposited onto a paper electrode. In the presence of the toxin, the peptide is cleaved, and voltammetric signal decreases. SWV, square wave voltammetry. Adapted with permission from [172].

Peptides are also an attractive alternative to antibodies in electrochemical biosensors, since they exhibit good affinity to specific targets and can be easily synthetized and chemically modified. Compared to antibodies, peptides show higher stability to temperature, pH, and ionic strength, and have longer shelf life and lower cost. Peptides, due to high variety of their amino acid composition and stability of their secondary structure, have remarkable recognition flexibility. In the last decade, various biological and chemical techniques for the rapid screening of peptide libraries identified various synthetic peptides that were used for biosensor development. Peptides are not redox active molecules and produce no measurable electrochemical signal directly in response to a binding event. However, peptide structure can be easily conjugated with a redox indicator, such as ferrocene, to enable direct conversion of the analyte detection into a measurable signal [171]. We recently reviewed peptide-based biosensors for foodborne pathogen detection [28]. Peptide-based electrochemical biosensors were reported for a broad spectrum of other targets including proteins, antibodies, toxins, DNA, and metallic ions. Gold electrodes are mostly used in peptide-based sensors because peptides can be efficiently immobilized using thiol–gold chemistry based on SAM methods. Moreover, some peptides can functionalize 2D nanomaterials, such as carbon nanotubes, graphene, and other semiconductors, by self-assembling controlled by noncovalent bonds. For instance, an electrochemical biosensor for the detection of botulinum neurotoxins produced by the soil bacterium *Clostridium botulinum* employed the methylene blue labeled peptide [172]. The peptide was immobilized on electrode carrying AuNPs by drop casting. In the presence of the botulinum neurotoxins, the peptide was cleaved, leading to the decrease of the signal due to the removal of methylene blue from the electrode surface (Figure 7). The electrode combined with a portable potentiostat and smartphone detected botulinum neurotoxins with a LOD of 10 pM in spiked samples of orange juice [173].

Direct application of peptide-based biosensors in foods may be impeded by the presence of peptidase and their degradation. The most reactive proteases in fresh and unprocessed foods are thermolysine, carboxypeptidases, and trypsin. Heating of the food sample prior to analysis may result in deactivation of proteases without the target alternation. For instance, this pretreatment can be applied when thermostable toxins are targeted by peptide-based biosensors. Alternatively, peptides can be chemically modified to make them resistant to proteolytic degradation. Lorenzon et al. [174] used click chemistry to induce peptide dimerization and multimerization in order to increase their stability. Specific molecules like sugars or fatty acids can be conjugated to N-, C-terminal, or side-chains, to make peptide cleavage sites inaccessible to the enzyme [175,176]. Nevertheless, there is no universal chemical modification that can be applied in peptide-based biosensors because structural modification may modify its recognition efficiency.

Molecularly imprinted polymers have been developed to overcome such limitations in bioreceptor stability. MIPs are fabricated by a monomer polymerization in the presence of target entities, which is extracted after the polymerization. In this way, the target serves as a template to generate selective cavities. Using various templates, polymers, and crosslinking agents and varying their ratios enable the formation of cavities with size and shape highly complementary to the target [177]. Besides being produced at low cost, MIPs have remarkable physical and chemical resistance, with affinity and selectivity toward targets comparable to natural receptors [178]. Tailor-made synthetic MIP receptors have been employed to produce stable, robust, and cheap recognition elements of target analytes in food, such as pesticides, veterinary drugs, mycotoxins, viruses, and bacterial cells [173].

The natural polymer chitosan was employed to quantify antioxidant catechol in wine [179]. MIP was formed using chitosan-encapsulated AuNP-decorated multiwalled carbon nanotubes matrix in the presence of catechol on a boron-doped diamond electrode. The voltammetric response of the sensor showed excellent reproducibility and repeatability to catechol detection in the range of 0 to 1 mM, with a LOD of 3.7 × 10^−5^ M. In another study, the MIP-based sensor for food allergen detection was shown to detect soy genistein in foods and resolve genistein from other structurally analogous isoflavones and flavones [100]. The analytical performances of the sensor were demonstrated in a range of solid and liquid foods, having over 100 different ingredients. The synthesis of MIPs for small molecular targets such as pesticides, antibiotic traces, or allergens is now well established. However, the production of MIPs with the much larger cavities that recognize whole microorganisms remains a challenge.

**Figure 8:**
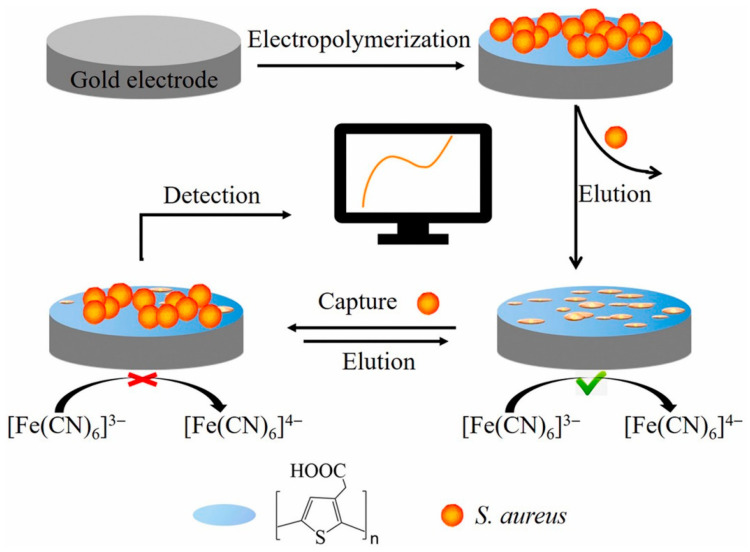
Schematic illustration for the fabrication of MIP-based impedimetric sensor for *S. aureus* detection, adapted with permission from [42].

Electronically conductive polymer 3-thiopheneacetic acid (TAA) was applied as a functional monomer for imprinting whole cells of *S. aureus*, which produced micrometer-sized cavities (Figure 8). This MIP receptor was formed directly on gold electrode surface using one-step electropolymerization and subsequent template elution [42]. Electrodeposition as a simple and fast technique allows the deposition of very porous films in a controlled manner. Under the optimized conditions, *S. aureus* was detected in contaminated milk samples within 10 min with a very low LOD of 2 CFU/mL and wide linear range from 10 to 10^8^ CFU/mL. The affinity of MIPs for whole bacterial cell detection can be improved by monomer modifications. For instance, it was shown that phenylboronic acid improves nonspecific adhesion of bacterial cells to the polymers but also creates specific interaction with cis-diol groups present on the bacterial surface [180].

Despite substantial progress in the development of MIP-based electrochemical sensors for food safety, this technology is still in its beginning. Many challenges related to nonspecific binding, incomplete removal of templates, and undesired adsorption have to be overcome. For instance, nonspecific binding of molecules from food matrix can plague detection of diluted targets. Moreover, the synthesis of some MIPs requires organic solvents that represents an environmental problem. Recent studies pointed out that MIPs show limited selectivity and mass transport ability [181,182,183]. Finally, it is still difficult to adapt MIP sensors for the detection of multiple analytes in food matrices.

### 2.4. Other Electrochemical Biosensors

Phages and phage receptor binding proteins are attractive recognition elements in biosensors for bacterial detection because they target whole cells and require no special sample preparation step, which significantly simplify the analysis. In addition, phage can be genetically modified to replace its native sequence of the receptor binding protein by sequences encoding for different peptides in order to modify the phage binding affinity. Shin et al. [184] performed phage display using two M13 phage libraries with cyclic and linear form of peptides to select peptides capable of binding specifically to food allergen ovomucoid. Selected whole phage viral particles were attached to a SAM-functionalized gold electrode using crosslinking chemistry. The cyclic peptide-displayed phage sensor exhibited significantly better analytical properties than linear phage sensor with LOD of 0.12 μg/mL for ovomucoid. The sensor was successfully applied for allergen detection in egg and white wine samples.

Bacteriophages have been also used as elements in biosensors for their lytic activity. Interaction of a lytic phage with the target bacterium causes bacterial cell lysis and liberation of intracellular enzymes that can be electrochemically detected. Another approach relies on genetic modification of the native bacteriophage by inserting genes encoding for exogeneous enzyme. Upon infection, the inserted enzyme is expressed in the living bacterial cell of the host. El-Moghazy et al. [31] engineered *E. coli*-targeting bacteriophage T7 by inserting a gene encoding alkaline phosphatase expression into its genome. A portable sensing platform was developed by association of this engineered bacteriophage with a disposable single-walled carbon nanotube modified screen-printed electrode. During infection of *E. coli* with the engineered phage, intracellular alkaline phosphatase is released. The enzyme was detected using its nonelectroactive substrate 1-naphthyl phosphate which hydrolyzed into the electroactive 1-naphthol, which is easily detectable. When the sensitivity of *E. coli* detection was investigated in spinach leaf samples, a very low bacterial concentration of 1 CFU/mL was detected within 7 h.

Electrochemical enzymatic biosensors couple the catalytic activity of enzymes and the electrochemical signal generation. Enzymes catalyze specific reactions, which may lead to the production or consumption of electroactive species or to electron transfer, which can then be electrochemically detected. The resulting electrical signal is proportional to the concentration of the target analyte, allowing for accurate quantification without the need of a redox probe. Enzymatic electrochemical biosensors have considerably evolved since the initial use of the glucose oxidase enzyme as a sensing element in a device that turned a therapeutic concentration of glucose into a digital signal [185]. Enzymes, being proteins, can be immobilized on the electrode surface through chemical and physical interactions ranging from classical adsorption, covalent binding, entrapment, cross-linking, or affinity [186]. The choice of the immobilization method represents an important factor that influences structure and catalytic activity of the enzyme and thus affects the sensor stability, sensitivity, and selectivity. Enzymatic biosensors are highly sensitive because of the enzymatic amplification produced.

De Brito et al. [99] reported an electrochemical quantification of lactose in skimmed milk using enzyme lactase. Milk is of great importance in human nutrition, as it is a source of calcium. To enable lactose-intolerant people to consume milk, the dairy industries are investing in the production of milk and dairy products with reduced content of lactose or zero lactose. Lactase enzyme was immobilized by adsorption onto the carbon paste electrode modified with carbon nanotubes. The LOD of the electrochemical biosensor for lactose was 0.15 mmol/L and showed high stability and strong repeatability. Therefore, the proposed biosensor, allowing a reliable and quick monitoring of lactose, is extremely promising for the dairy sector and consumers.

Tyrosine is one of the essential amino acids necessary to support nutritional balance, and its level in the body indicates a person’s health status closely related to the consummated food. Varmira et al. [140] developed an enzymatic electrochemical biosensor for monitoring of tyrosine in food samples. For this, tyrosine hydroxylase enzyme was immobilized onto palladium–platinum bimetallic alloy nanoparticles/chitosan-1-ethyl-3methylimidazolium bis(trifluoromethylsulfonyl) imide/graphene-multiwalled carbon nanotubes-IL/glassy carbon electrode. Immobilization of the enzyme was achieved by cross-linking tyrosine hydroxylase and chitosan with glutaraldehyde. The electrode was applied for quantification of L-tyrosine in some high tyrosine foods like cheese, egg and yogurt and showed an LOD of 9 × 10^−12^ mol/L.

**Figure 9:**
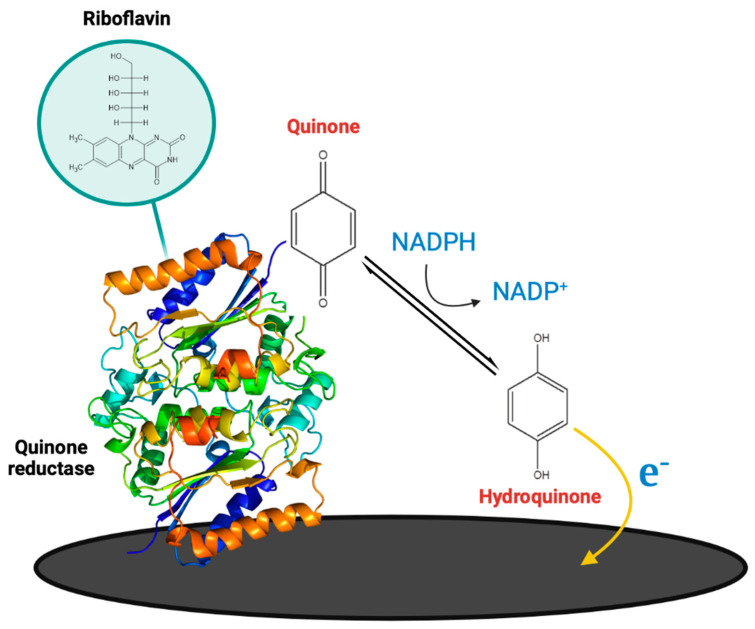
Schematic illustration of the carbon screen printed electrode modification with quinone reductase enzymatic biosensor construction and detection of vitamin K_3_ in solution containing NADPH as an electron donor and riboflavin.

We recently developed an electrochemical biosensor based on the NADPH-dependent quinone reductase enzyme for rapid and redox probe-free detection of vitamin K_3_ [187]. Vitamin K is a group of vitamins that play an important role in blood coagulation but their excess may cause severe side effects. The enzyme was immobilized onto the disposable carbon screen printed electrode using the drop-casting method. When vitamin K_3_ was added to the electrode, quinone reductase reduced it to the hydroquinone form in the presence of NADPH and riboflavin. Formed hydroquinone oxidized on the electrode surface, generating a specific and strong electrochemical signal (Figure 9). The practical potential of the biosensor, when tested in spiked milk samples, achieved 15-min quantification of the vitamin K_3_ with an LOD of 0.86 μM.

Many challenges in this type of electrochemical biosensors still need to be resolved, including the high expense of enzyme production and purification, lack of reliable responses at low concentrations, interference reactions, and the stability of the enzymes, which can be denatured by various factors such as temperature, pH, and food matrix component.

## 3. Conclusions and Perspectives

In this review, we have presented the advances in electrochemical biosensors from the last few years with an emphasis on the concept of assays. In the field of food analysis, the development of biosensors has changed the safety control practice and procedures for the detection of contaminants. As outlined electrochemical biosensors may simplify procedure and significantly reduce time, cost and reagent consumption of analysis compared to traditional and molecular methods. Since the first commercialization of glucose electrochemical biosensors, huge efforts have been made in the development of new electrochemical designs for other analytes such as pathogens, antibiotics, heavy metals, allergens, and pesticides. Enzymes are being substituted with less expensive and more resistant bioreceptors such as aptamers, peptides, DNA probes, or MIP that are less dependent on reaction media and thus more adapted for direct measurements in food matrices. For the detection of a low-concentration contaminant in food, electrochemical biosensors are designed for specific bioreceptors, signal amplifiers, and electrode surface modifications to increase specific surface and its electroconductivity. Notable advances have been made to enhance the sensitivity, enable evaluation of complex samples, decrease the price, and provide ease of operation and rapid time to result.

The future trends and challenges concerning electrochemical biosensor for food analysis will include the development of new types of low-cost, biocompatible, and eco-friendly electrodes, innovative engineering of bioreceptors, and application of novel functional nanomaterials for signal enhancement together with further device miniaturization to handheld and multiplex format. Such disposable sensors may be integrated with blockchain technologies for quality control along a production and supply food chain. To address such a complex challenge, cooperation among diverse researchers with professional food control analysts is needed. This would compensate for the need for expensive traditional testing and offer significant benefits.

## Figures and Tables

**Table 1:** List of recent publications of electrochemical biosensors for food safety and control.

Target	Technique	Bioreceptor	Electrode	Lod	Redox Indicator	Reference
*E. coli*	Osteryoung square wave voltammetry (OSWV)	Antibodies	Graphite felt electrode	400 cells/mL	TMB	[29]
*E. coli*	Electrochemical impedance spectroscopy (EIS)	Bioorthogonal conjugation reaction	Gold electrode	12 CFU/mL	Ferri/Ferrocyanide	[30]
*E. coli*	Differential pulse voltammetry (DPV)	Phages	Screen-printed carbon electrode	1 CFU/mL	Ferri/Ferrocyanide	[31]
*E. coli*	Square wave voltammetry (SWV)	DNA	Gold coated electrode	0.01 zM	Toluidine blue	[32]
*E. coli*, *S. aureus*	DPV	Enzyme	Gold coated electrode	100 CFU/mL	/	[33]
*Listeria*	EIS	Aptamers	Platinum–iridium electrode	3.3 CFU/mL	Hydroquinone, Hexaamineruthenium(III) chloride, Potassium ferrocyanide trihydrate	[34]
*Salmonella*	DPV	Antibodies	Glassy carbon electrode	1.2 × 10^2^ CFU/mL	Ferri/Ferrocyanide	[35]
*S. typhimurium*	EIS	Antibodies	Interdigitated microelectrode	19 CFU/mL	MnO_2_/H_2_O_2_	[36]
*S. typhimurium*	EIS	Antibodies	Interdigitated microelectrode	10^1^ CFU/mL	MnO_2_/H_2_O_2_	[37]
*S. typhimurium*	EIS	Antibodies	Glassy carbon electrode	35 CFU/mL	Ferri/Ferrocyanide	[38]
*Salmonella*	DPV	Antibodies	Glassy carbon electrode	2.4 × 10^2^ cfu/mL	Ferri/Ferrocyanide	[39]
*S. typhimurium*	DPV	Aptamers	Screen-printed carbon electrode	16 CFU/mL	Methylene blue	[40]
*S. aureus*	DPV	Aptamer + dsDNA	Gold electrode	8 CFU/mL	Ferri/Ferrocyanide	[41]
*S. aureus*	EIS	Antibodies	Gold electrode	2 CFU/mL	Ferri/Ferrocyanide	[42]
*S. aureus*	DPV	Antibodies	Gold electrode	28.55 CFU/mL	Potassium ferricyanide	[43]
*S. aureus*	Electrochemiluminescence (ECL)	Aptamers	Glassy carbon electrode	3 CFU/mL	Tris (2,2-bipyridyl) dichlororuthenium (II) hexahydrate	[44]
*S. aureus*	DPV	Aptamers + ssDNA	Gold electrode	9 CFU/mL	Methylene blue	[45]
*S. aureus*	DPV	Antibodies	Gold disk electrode	2 CFU/mL	Ag_2_S/HNO_3_	[46]
*S. aureus*	CV	DNA	Gold electrode	2.37 × 10^5^ U/μL	Ferri/Ferrocyanide	[47]
*S.aureus*	SWV	SRCA-CRISPR (DNA)	Glassy carbon electrode	3 CFU/mL	Methylene blue	[48]
*S. aureus*	Chronoamperometry	Aptamers	Gold electrode	3 CFU/mL	Tyramide/Horseradish peroxidase/H_2_O_2_	[49]
*S. aureus*	DPV	Antibodies	Gold electrode	0.04 ng/mL	Silver nanoparticles	[50]
*S. aureus*	DPV	Aptamers	Glassy carbon electrode	1 CFU/mL	Methylene blue	[51]
*Shigella flexneri*	DPV	SRCA-CRISPR (DNA)	Indium tin oxide electrode	10 cells/ml	Ferri/Ferrocyanide	[52]
*B. cereus*	SWV	Antibodies	Glassy carbon electrode	10^1^ CFU/mL	Potassium ferricyanide	[53]
*Aphanizomenon* *flos-aquae*	CV, EIS	DNA	Gold electrode	81.2 pg/mL 9.99 pg/mL	Ferri/Ferrocyanide	[54]
*V. parahaemolyticus*	DPV	Aptamers	Screen-printed carbon electrode	5.74 CFU/mL	Ferri/Ferrocyanide	[55]
Norovirus	DPV	Aptamers	Screen-printed carbon electrode	0.28 ng/mL	Ferri/Ferrocyanide	[56]
*Multi bacteria*	EIS	Peptides	Gold electrode	10^1^ CFU/mL	Ferri/Ferrocyanide	[57]
*E. coli*, *S. aureus*, and *S. typhimurium*	DPV	Aptamers	Screen-printed gold electrode	3 CFU/mL	Methylene blue	[58]
Ochratoxin A	DPV	Enzymes + CRISPR-Cas12a	Glassy carbon electrode	0.74 fg/mL	Ferrocene	[59]
Ochratoxin A	DPV	DNA tetrahedron	Gold electrode	0.773 pg/mL	AgNCs/H_2_O_2_	[60]
Ochratoxin A	DPV	Aptamers	Glassy carbon electrode	0.05 pg/mL	Methylene blue	[61]
Ochratoxin A	SWV	Aptamers	Glassy carbon electrode	81 fg/mL	Ferrocene, Methylene blue	[62]
Ochratoxin A	DPV	Aptamers	Gold electrode	5 pM	Ferri/Ferrocyanide	[63]
Ochratoxin A	EIS	Aptamers	Screen-printed carbon electrode	1 pg/mL	Potassium hexacyanoferrate (III)	[64]
Ochratoxin A	DPV	Aptamers	Gold electrode	0.289 pg/mL	Tetraferrocene	[65]
Ochratoxin A	DPV	Aptamers	Gold electrode	6.79 fg/mL	Horseradish peroxidase/H_2_O_2_	[66]
Ochratoxin A	SWV	Aptamers	Gold electrode	0.235 pg/mL	Ferrocene, Methylene blue	[67]
Ochratoxin A	DPV	Aptamers	Gold electrode	1.12 fg/mL	Methylene blue	[68]
Aflatoxin B1	DPV	Aptamers	Gold electrode	2.84 fg/mL	Methylene blue	[69]
Aflatoxin B1	DPV	Aptamers	Glassy carbon electrode	1.82 pg/mL	Ferri/Ferrocyanide	[70]
Aflatoxin B1	EIS	Aptamers	Titanium oxide nanotube arrays electrode	1 pg/mL	Hexacyanoferrate	[71]
Aflatoxin B1	DPV	Antibodies	Glassy carbon electrode	0.18 ng/mL	Potassium ferricyanide	[72]
Aflatoxin B1	DPV	Aptamers	Screen-printed carbon electrode	15.16 ag/mL	Ferri/Ferrocyanide	[73]
Aflatoxin B1	EIS	Antibodies	Screen-printed carbon/graphite electrode	0.092 ng/mL	Ferri/Ferrocyanide	[74]
Aflatoxin B1	DPV	Antibodies	Carbon fiber microelectrode	8 pg/mL	Potassium ferricyanide	[75]
Aflatoxin B1	Alternating Current Voltammetry (ACV)	Aptamers	Glassy carbon electrode	38.8 pg/mL	Ferrocene, Methylene blue	[76]
Aflatoxin B1	DPV	Aptamers	Indium tin oxide electrode	0.032 pg/mL	Ferrocene, Methylene blue	[77]
Aflatoxin B1	DPV	Aptamers	Glassy carbon electrode	0.5 pM	Methylene blue	[78]
Aflatoxin B1	SWV	Aptamers	Glassy carbon electrode	0.12 pM	Ferrocene, Methylene blue	[79]
Aflatoxin B1	EIS	Aptamers	Screen-printed carbon electrode	0.01 nM	Potassium ferricyanide	[80]
Aflatoxin M1	Chronoamperometry	Antibodies	Screen-printed carbon electrode	0.09 ng/mL	Ferri/Ferrocyanide	[81]
Aflatoxin M1	EIS	Aptamers	Pencil graphite electrode	0.3 ng/L	Ferri/Ferrocyanide	[82]
Aflatoxin M1	DPV	Aptamers	Gold electrode	0.02 ng/mL	Methylene blue	[83]
Total Aflatoxins	Chronoamperometry	Antibodies	Screen-printed carbon electrode	0.017 μg/L	TMB	[84]
Total Aflatoxins	DPV	Antibodies	Glassy carbon electrode	0.05 pg mL	Potassium ferricyanide	[85]
Deoxynivalenol	DPV	Aptamers	Glassy carbon electrode	0.008 ng mL	N-doped Cu-metallic organic framework	[86]
Deoxynivalenol	SWV	Aptamers	Gold electrode	6.9 × 10^−9^ mg/mL	Methylene blue	[87]
T-2	DPV	Aptamers	Gold electrode	0.107 fg/mL	Methylene blue	[88]
T-2	DPV	Aptamers	Gold electrode	8.74 × 10^−7^ ng/mL	Methylene blue	[89]
Patulin	DPV	Aptamers	Gold electrode	4.14 × 10^−5^ ng/mL	Methylene blue	[90]
Patulin	SWV	Aptamers	Glassy carbon electrode	0.043 nM	Ferrocene monocarboxylic acid, Methylene blue	[91]
Patulin	DPV	Aptamers	Gold electrode	0.217 fg/mL	Thionine	[92]
Staphylococcal Enterotoxin A	DPV	Aptamers	Screen-printed carbon electrode	7.6 fM	Hematoxylin	[93]
Zearalenone	DPV	Aptamers	Gold electrode	5 fg/mL	Methylene blue, Ag^+^	[94]
Zearalenone	EIS	Aptamers	Gold electrode	7 fg/mL	Ferri/Ferrocyanide	[95]
Zearalenone	DPV	Aptamers	Glassy carbon electrode	1.0 × 10^−5^ ng/mL	Toluidine blue	[96]
Fumonisin B1	DPV	Aptamers	Gold electrode	0.306 fg/mL	Methylene blue	[97]
Endotoxins	DPV	Aptamers	Glassy carbon electrode	0.55 fg/mL	Ferri/Ferrocyanide	[98]
Lactose	CV	Enzyme	Carbon electrode	0.15 mmol/L	/	[99]
Soy	DPV	Molecularly imprinted polymers	Polymer-based screen-printed electrode	100 ppb	Ferri/Ferrocyanide	[100]
Gluten	EIS	Aptamers	Screen-printed carbon electrode	0.05 mg/L	Ferri/Ferrocyanide	[101]
Tropomyosin	Linear sweep voltammetry (LSV)	Antibodies	Screen-printed carbon electrode	0.47 ng/mL	Indigo blue/Ag^+^	[102]
Ara h 1/Ara h 6	LSV	Antibodies	Screen-printed carbon electrode	5.2 ng/mL and 0.017 ng/mL	Ag^+^	[103]
Ara h 1	DPV	Aptamers	Screen-printed carbon electrode	1.66 nM	Ferrocene Dimethanol	[104]
Ara h 1	DPV	Aptamers	Screen-printed carbon electrode	21.6 ng/mL	Ferri/Ferrocyanide	[105]
Penicillin	DPV	Aptamers	Screen-printed carbon electrode	0.05 nM	Ferri/Ferrocyanide	[106]
Arsenic	Square wave stripping voltammetry (SWSV)	NPs	Screen-printed carbon electrode	16.73 μg/L	Potassium ferricyanide	[107]
Carbendazim, Diuron, Paraquat, Fenitrothion	DPV	NPs	Glove-embedded screen-printed carbon electrode	4.7 × 10^−8^; 9.2 × 10^−7^; 2.4 × 10^−8^; 6.4 × 10^−7^	Ferri/Ferrocyanide	[108]
Carbendazim	DPV	Aptamers	Screen-printed carbon electrode	4.35 nM	Ferri/Ferrocyanide	[109]
Fenhexamid	SWV	NPs	Glassy carbon electrode	1.32 μmol/L	BRB/methanol	[110]
Quinolones	EIS, CV	DNA	Gold electrode	0.052 ng mL	Methylene blue	[111]
Nickel ions	LSV, CV	Synthetic receptors	Screen-printed carbon electrode	0.005 mg/L	Nickel ions	[112]
Pb^2+^	SWV	3d DNA nanostructure	Glassy carbon electrode	2.61 pM	Methylene blue	[113]
Pb^2+^	DPV	Aptamers	Glassy carbon electrode	0.33 ng/L	Ferri/Ferrocyanide	[114]
Pb^2+^ and Cd^2+^	SWV	Aptamers	Gold electrode	89.31 pmol/L; 16.44 pmol/L	Ferrocene, Methylene blue	[115]
Acrylamide	DPV	Aptamers	Glassy carbon electrode	0.104 nM	Ferri/Ferrocyanide	[116]
Ampicillin	DPV	Antibodies	Indium tin oxide electrode	0.028 μg/mL	Ferri/Ferrocyanide	[117]
Parathion	CV	Antibodies	Screen-printed carbon electrode	2.26 pg/mL	HQ/HRP/H_2_O_2_ system	[118]
Nitrofurans	SWV	Antibodies	Gold electrode	1.35 × 10^−7^ μg/L	Methylene blue	[119]
Tiamulin	DPV	Antibodies	Gold electrode	0.003 ng/mL	Ferri/Ferrocyanide	[120]
Rhodamine B	CV	Antibodies	Screen-printed carbon electrode	0.89 pg/mL	Cerium oxide	[121]
Carbaryl	Linear scan anodic stripping voltammetry (LSASV)	Antibodies	Gold-graphite electrode	0.08 μg/kg	Copper conjugate label	[122]
Enrofloxacin	DPV	Antibodies	Screen-printed dual carbon electrode	0.003 μg/mL	Aminoferrocene	[123]
Sulfamethazine	DPV	Aptamers	Glassy carbon electrode	4.0 pM	Hexaamineruthenium(III) chloride	[124]
Melamine	DPV	Aptamers	Glassy carbon electrode	6.7 × 10^−13^ M	Ferri/Ferrocyanide	[125]
Streptomycin	DPV	Aptamers	Gold electrode	0.0033 nM	Methylene blue	[126]
Streptomycin	DPV	Aptamers	Glassy carbon electrode	2.31 nM	Ferri/Ferrocyanide	[127]
Streptomycin	DPV	Aptamers	Gold electrode	0.003 nM	Ferri/Ferrocyanide	[128]
Chloramphenicol	DPV	Aptamers	Gold electrode	2.08 pmol/L	Ferri/Ferrocyanide	[129]
Chloramphenicol	DPV	Aptamers	Glassy carbon electrode	0.03 pM	Ferri/Ferrocyanide	[130]
Bisphenol A	DPV	Aptamers	Gold electrode	3.65 pM	Ferri/Ferrocyanide	[131]
Tetracycline	DPV	Aptamers	Glassy carbon electrode	4.8 × 10^−2^ nM	Ferri/Ferrocyanide	[132]
Tetracycline	DPV	Aptamers	Glassy carbon electrode	3.2 × 10^−16^ M	Ferri/Ferrocyanide	[133]
Tetracycline	DPV	Aptamers	Glassy carbon electrode	2.28 × 10^−18^ M	Ferri/Ferrocyanide	[134]
Di(2-ethylhexyl) phthalate	DPV	Aptamers	Gold electrode	0.04 ng/mL	Methylene blue	[135]
Paraquat	DPV	Aptamers	Glassy carbon electrode	0.34 g/L	Nickel hexacyanoferrate nanoparticles	[136]
Thiamethoxam	DPV	Aptamers	Glassy carbon electrode	3.65 × 10^−3^ μg/L	Nickel hexacyanoferrate nanoparticles	[137]
Progesterone	DPV	Aptamers	Glassy carbon electrode	1.73 × 10^−15^ M	Ferri/Ferrocyanide	[138]
Malathion and Omethoate	ACV	Aptamers	Glassy carbon electrode	1.3 pg/mL; 2.8 pg/mL	Ferrocene, Methylene blue	[139]
Tyrosin	EIS	Enzyme	Glassy carbon electrode	9 × 10^−12^ mol/L	/	[140]
Acetoin	EIS	Enzyme	Capacitive electrolyte-insulator-semi-conductor	Not mention	/	[141]
Organophosphorus pesticides	EIS	Enzyme	Glassy carbon electrode	2.78 × 10^−11^ g/L	/	[142]
Organophosphorus pesticides	EIS	Enzyme	Gold electrode	0.1 and 1.5 nM	/	[143]
Glyphosate	EIS	Enzyme	Screen-printed carbon electrode	0.015 μg/mL, 0.045 μg/mL	/	[144]
Malathion, Trichlorfon	Amperometry	Enzyme	Glassy carbon electrode	0.032 μg/L Malathion; 0.001 μg/L trichlorfon	/	[145]
Organophosphorus pesticides	EIS	Enzyme	Glassy carbon electrode	7.4 nM	/	[146]
Soybean	EIS	DNA	Gold electrode	1.792 ng/mL	Ferri/Ferrocyanide	[147]
Soybean	ECL, Fast Scan Voltammetry (FSV)	Cas12a (Cas protein family)	Magnetic glass carbon electrode	0.3 fmol/L (ECL); 3 fmol/L (FSV)	Ferrocene, Bis(2,2′-bipyridine)-(5-aminophenanthroline)ruthenium bis(hexafluorophosphate)	[148]
GM crops	ECL	Antibodies	Magnetic glass carbon electrode	0.001 ng/mL	PTCA and S_2_O_8_^2−^	[149]
